# Common basis for orofacial clefting and cortical interneuronopathy

**DOI:** 10.1038/s41398-017-0057-7

**Published:** 2018-01-10

**Authors:** Lydia J. Ansen-Wilson, Joshua L. Everson, Dustin M. Fink, Henry W. Kietzman, Ruth Sullivan, Robert J. Lipinski

**Affiliations:** 1 Department of Comparative Biosciences, School of Veterinary Medicine, University of Wisconsin-Madison, 2015 Linden Drive, Madison, WI 53706 USA; 2 Comparative Biomedical Sciences Graduate Program, School of Veterinary Medicine, University of Wisconsin-Madison, 2015 Linden Drive, Madison, WI 53706 USA; 3 Molecular and Environmental Toxicology Center, School of Medicine and Public Health, University of Wisconsin-Madison, 1010B McArdle Building, 1400 University Avenue, Madison, WI 53706 USA

## Abstract

Orofacial clefts (OFCs) of the lip and/or palate are among the most common human birth defects. Current treatment strategies focus on functional and cosmetic repair but even when this care is available, individuals born with OFCs are at high risk for persistent neurobehavioral problems. In addition to learning disabilities and reduced academic achievement, recent evidence associates OFCs with elevated risk for a constellation of psychiatric outcomes including anxiety disorders, autism spectrum disorder, and schizophrenia. The relationship between these outcomes and OFCs is poorly understood and controversial. Recent neuroimaging studies in humans and mice demonstrate subtle morphological brain abnormalities that co-occur with OFCs but specific molecular and cellular mechanisms have not been investigated. Here, we provide the first evidence directly linking OFC pathogenesis to abnormal development of GABAergic cortical interneurons (cINs). Lineage tracing revealed that the structures that form the upper lip and palate develop in molecular synchrony and spatiotemporal proximity to cINs, suggesting these populations may have shared sensitivity to genetic and/or teratogenic insult. Examination of cIN development in a mouse model of nonsyndromic OFCs revealed significant disruptions in cIN proliferation and migration, culminating in misspecification of the somatostatin-expressing subgroup. These findings reveal a unified developmental basis for orofacial clefting and disrupted cIN development, and may explain the significant overlap in neurobehavioral and psychiatric outcomes associated with OFCs and cIN dysfunction. This emerging mechanistic understanding for increased prevalence of adverse neurobehavioral outcomes in OFC patients is the entry-point for developing evidence-based therapies to improve patient outcomes.

## Introduction

Orofacial clefts (OFCs) are frequently occurring human birth defects that have a complex, multifactorial etiology^1,2^. Though structural orofacial defects are often surgically corrected during infancy and early childhood, OFC patient cohorts are at high risk for neurobehavioral problems including learning disability, impaired language function, psychosocial adjustment issues, and persistently reduced academic achievement^3–15^. More recently, OFCs have been associated with significantly increased risk for a constellation of psychiatric-related outcomes including anxiety disorders, autism spectrum disorders, epilepsy, and schizophrenia^15,16^. Though these neurobehavioral problems contribute significantly to the morbidity associated with OFCs, they are not currently addressed by standard treatment plans, in part because causative factors remain controversial and poorly understood.

The etiological bases proposed for OFC-associated learning and neurobehavioral problems can be divided into two categories: primary neurodevelopmental disruptions and secondary postnatal influences. Postnatal environmental influences such as surgical procedures, anesthesia, and social stigma have long been presumed to be the most significant effectors but supportive evidence is limited. For example, recent examination of a large population of patients with OFCs found that academic performance was dependent upon the type of cleft but independent of the number and timing of anesthesia events and operations^17^. An emerging alternative hypothesis holds that OFC-associated adverse neurobehavioral outcomes stem from primary neuroanatomical abnormalities related to cleft pathogenesis itself. In addition to the well-described interdependence of face and brain development^18^, this hypothesis is supported by recent neuroimaging studies documenting subtle and partially overlapping structural brain anomalies in patients born with OFCs^19–23^, in fetal mice with OFCs^24^, and adult mice with OFC-associated mutations^25^.

Cellular and molecular CNS alterations accompanying OFCs, however, have not previously been examined in human populations or animal models. Addressing this fundamental gap, we show here in the mouse that nonsyndromic OFCs can co-occur with significant disruptions in GABAergic cortical interneuron (cIN) development. Though they comprise only 10–25% of neurons in the neocortex, the dynamic modulation of cortical activity exerted by cINs shapes cortical maturation, underlies multiple aspects of learning and memory, and is requisite for normal cognition^26,27^. Disruption of cIN development has been extensively implicated in neurodevelopmental and neuropsychiatric illnesses that have symptomatic overlap with traits observed in populations with OFCs^26,28–36^. Our findings are the first to link OFCs with abnormal cIN development and represent an important foundational step in understanding neurobehavioral deficits that contribute significantly to the morbidity of common birth defects.

## Materials and methods

### Animal studies

All experiments were carried out in strict accordance with the recommendations set forth in the National Institutes of Health’s Guide for the Care and Use of Laboratory Animals. Authorization for experiments was granted by the University of Wisconsin School of Veterinary Medicine Institutional Animal Care and Use Committee (protocol number G005396). C57BL/6J wildtype, Gli1^tm3(cre/ERT2)Alj^ (stock number: 007913), and B6.129S4-Gt(ROSA)26Sor^tm1Sor^ (stock number: 003474) mice were purchased from The Jackson Laboratories (Bar Harbor, ME). All mice were housed under specific pathogen-free conditions in disposable, ventilated cages in rooms maintained at 22±2 ^∘^C and 30–70% humidity on a 12-h light, 12-h dark cycle. Mice were fed 1919x Irradiated Harlan Teklad Global Soy Protein-Free Extruded Rodent Diet. Precise timed-pregnancies were established as previously described^37,38^ between 3–12-month-old male mice and 2–5-month-old females. Cyclopamine (LC Laboratories, Woburn, MA) was administered at 90–120 mg/kg/day starting at gestational day (GD8.25) to pregnant dams via continuous subcutaneous infusion using surgically implanted Alzet 2001D microosmotic pumps (Durect, Cupertino, CA) as previously described^39^. Mice were killed by CO_2_ asphyxiation and subsequent cervical dislocation. Fetal specimens were dissected in cold phosphate-buffered saline and fixed in 4% paraformaldehyde for *in situ* hybridization (ISH), 10% formalin for immunohistochemistry  (IHC) and histology, or Bouin’s solution for imaging and histology. Brightfield images were captured using a micropublisher 5.0 camera connected to a Nikon SZX-10 stereomicroscope. Confocal images were captured using a Leica SP8 confocal microscope.

### Gene expression analyses

Analyses were conducted on a previously published microarroarray data set^40^ (GEO accession number: GSE98336) to identify differentially expressed genes during the initial pathogenesis of orofacial clefting. For each treatment group, six litters were collected and microdissected frontonasal prominence (FNP) tissue from each embryo in the litter was pooled. Enrichment analyses of significant differentially expressed genes were conducted using PANTHER for the Gene Ontology term telencephalon^41^ or using large published gene sets for cIN^42^ and OFC^43^. Affymetrix Transcriptome Analysis Console was used for determination of significant differential expression in microarray experiments, with an FDR *P*-value of 0.75 (Benjamini−Hochberg). Enrichment analysis was conducted using the GeneProf hypergeometric probability calculator (https://www.geneprof.org/GeneProf/tools/hypergeometric.jsp).

### Lineage tracing

Lineage tracing was conducted as previously described^44^. Male Gli1^tm3(cre/ERT2)Alj^/J mice were mated to B6.129S4-Gt(ROSA)26Sortm1Sor/J female mice. Timed-pregnant females were administered a single dose of 25 mg/kg Tamoxifen at GD8.75 by IP injection. For each time point, multiple sections from at least three embryos from three independent litters were analyzed. Following dissection, embryos younger than GD12.0 were fixed in 4% paraformaldehyde on ice for 20 min and X-gal stained as described previously^44^. Embryos older than GD12.0 were fixed overnight in 2% paraformaldehyde with 0.2% glutaraldehyde. Tissues were post-fixed in 4% paraformaldehyde and either hemisected at the sagittal midline or embedded in 4% agarose and sectioned at 50 μm using a Leica VT1000A automatic vibrating blade microtome.

### Immunohistochemistry

Immunofluorescence staining was performed as previously described^45^. Primary antibodies were as follows: 1:125 chicken anti-beta galactosidase (ab9361, Abcam, Cambridge, MA), 1:1000 rabbit anti-GABA (A2052, Sigma, St. Louis, MO), 1:250 anti-Ki67 (#9027, Cell Signaling), 1:100 chicken anti-beta III Tubulin (ab107216, Abcam, Cambridge, MA). Secondary antibodies were diluted in RGBTw as follows: 1:250 goat anti-chicken Alexa Fluor 546 (A-11040, Thermo Fischer Scientific, Waltham, MA), 1:250 goat anti-rabbit DyLight 594 (35560, Thermo Fisher Scientific), 1:250 goat anti-mouse DyLight 594 (35511, Thermo Fisher Scientific), 1:250 goat anti-chicken Alexa Fluor 488 (A-11039, Thermo Fisher Scientific). For Ki67 quantification, a blinded rater processed each image to reduce background and optimize signal accuracy. Ki67-positive cells/area in each z-image were determined using the ImageJ (NIH) cell counter tool. For each treatment group, at least three embryos were examined from each of three litters. Values represent the mean±s.e.m.; ****P ≤ 0.0001, two-tailed Student’s *t-*test.

### RNA *in situ* hybridization

ISH was performed as previously described^40,44^ with probe sequences listed in Table S1. For each gene and stage, multiple sections from at least two embryos were analyzed from each of three independent litters.

### RNA isolation and real-time PCR

RNA extraction and purification, and real-time polymerase chain reaction (RT-PCR) were conducted as previously described^38,44^. For analysis of *Ccnd* expression levels at GD9.5, three individual litters were collected and embryos were pooled across a litter for each treatment group. Values represent the mean±s.e.m.; *P ≤ 0.05, two-tailed Student’s *t-*test. For analysis of GABAergic and glutamatergic markers, RNA was isolated from one cerebral hemisphere of vehicle exposed fetuses (*n* = 5 fetuses from two litters) and cyclopamine-exposed fetuses with overt upper lip clefts *n* = 7 fetuses from two litters); values represent the mean±s.e.m. *P ≤ 0.05, two-tailed Student’s *t-*test.

### MGE morphometric analysis

NIH ImageJ software was used to manually outline and capture the area of the medial ganglionic eminences (MGEs) and heads from brightfield images of hemisected GD11 vehicle- and cyclopamine-exposed embryos. The ratio of MGE area to total head area (as shown in Fig. S2) was then compared between exposure groups. Individual data points are shown with mean±s.d. of *n* = 24 embryos from three litters for the control group and *n* = 19 embryos from three litters for the OFC group; ****P ≤ 0.0001, two-tailed Student’s *t-*test.

### Measurement of cortical GABA levels

Cerebral cortices were microdissected from GD17 fetuses immediately following dissection. RNA was isolated from one cerebral hemisphere of vehicle exposed fetuses (*n* = 5 fetuses from two litters) and cyclopamine-exposed fetuses with overt upper lip clefts (*n* = 7 fetuses from two litters). Samples were homogenized using a tip sonicator (Misonix, Farmingdale, NY) and a centrifuged supernatant was prepared to assay GABA levels using a mouse gamma-aminobutyric acid (GABA) ELISA kit with a sensitivity of 0.1 μmol/L (Novatein Biosciences, Cambridge, MA) as previously described^46^. A Pierce BCA protein assay (Thermo Fischer Scientific) was used to determine the protein concentration of each supernatant. GABA concentration was then determined as ng GABA per mg protein. Values represent the mean±s.e.m. *P ≤ 0.05, two-tailed Student’s *t-*test.

## Results

### Expression profiling links OFC pathogenesis with cortical interneuron development

**Fig. 1:**
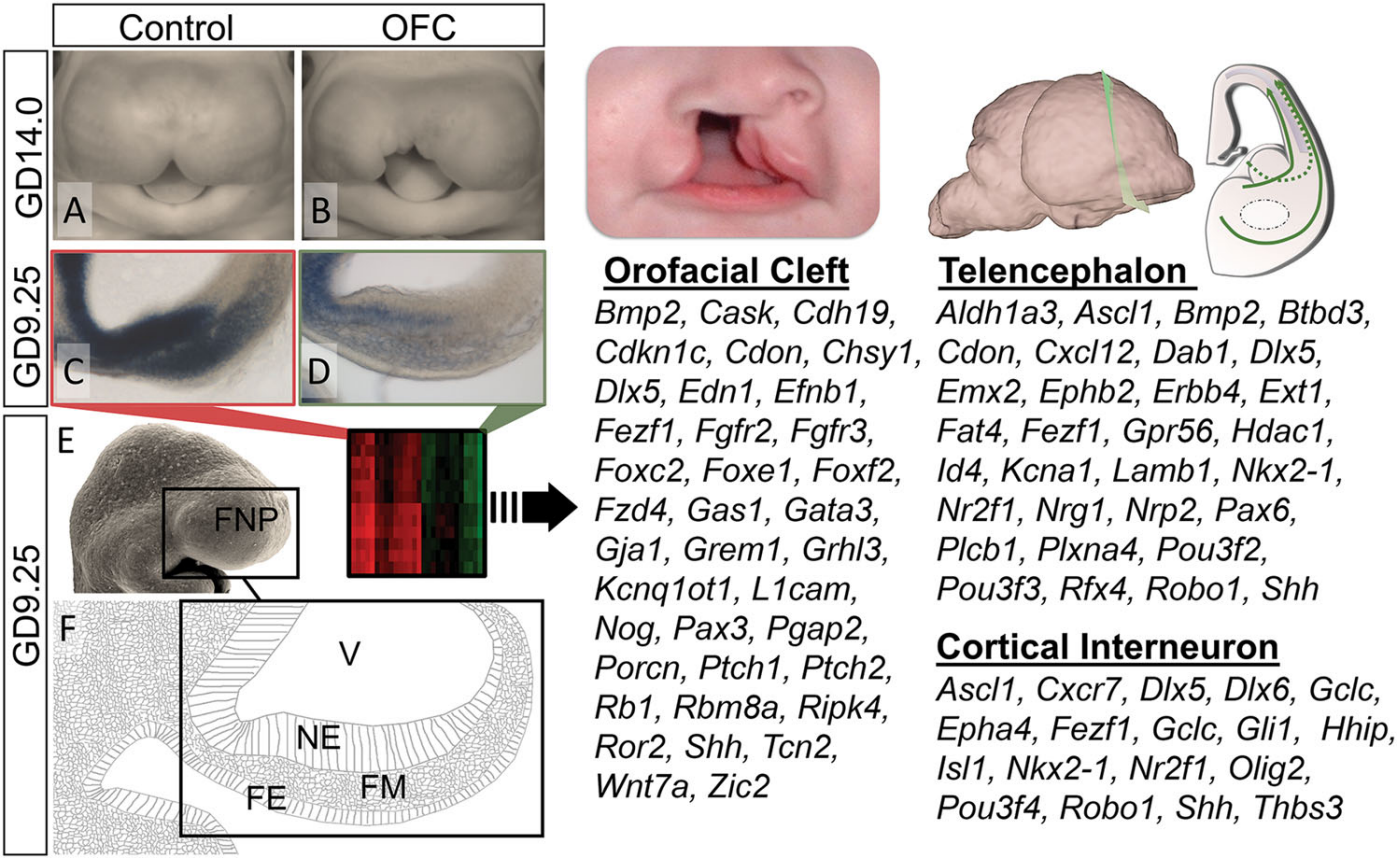
Changes in telencephalon and cortical interneuron transcripts during OFC pathogenesis. **a**,**b** Representative vehicle-exposed control and cyclopamine-exposed OFC embryos at GD14.0. **c**,**d** ISH staining for *Gli1* on parasagittal sections through the frontonasal prominence (FNP) of GD9.25 embryos illustrates reduced Shh-signaling activity during initial OFC pathogenesis. **e**,**f** Along with a scanning electron micrograph image of a GD9.25 mouse embryo, a schematic of a parasagittal section shows the cell populations comprising the FNP including the neuroectoderm (NE), facial mesenchyme (FM) and facial ectoderm (FE). (Right) Microarray analyses of microdissected FNP tissue from GD9.25 control versus OFC groups revealed differential expression of orofacial cleft, telencephalon, and cortical interneuron-related genes. V ventricle

Recent evidence implicating primary neurodevelopmental disruptions in the etiology of OFC-associated neurobehavioral traits prompted us to investigate molecular and cellular mechanisms underlying this relationship. We used an established mouse OFC model in which transient and temporally-specific administration of the  Sonic Hedgehog (Shh) signaling pathway inhibitor, cyclopamine, results in clefts of the lip that typically extend into the primary and secondary palate^47^ (Fig. 1a, b). To identify disrupted signaling networks and implicated neuronal populations, we analyzed a published data set^48^ of gene expression changes from microdissected tissue encompassing both the primordial midface and the adjacently developing prosencephalic neuroectoderm (Fig. 1c–f). First, we investigated the breadth of relevance of the experimental model to broader OFC pathogenesis by examining all genes with known OFC association^43^. Expression of more than 10% (37 of 362) of OFC genes was dysregulated in the OFC group relative to controls (Fig. 1). Further analyses identified a large number of genes related to telencephalon development, as expected. More surprising was an observed enrichment in genes related to cIN development. This suggested a potential relationship between OFC pathogenesis and development of the MGEs, bilaterally paired transitory telencephalic structures that give rise to a majority of cINs.

### Orofacial and cortical interneuron development occur in spatiotemporal proximity and molecular synchrony

**Fig. 2:**
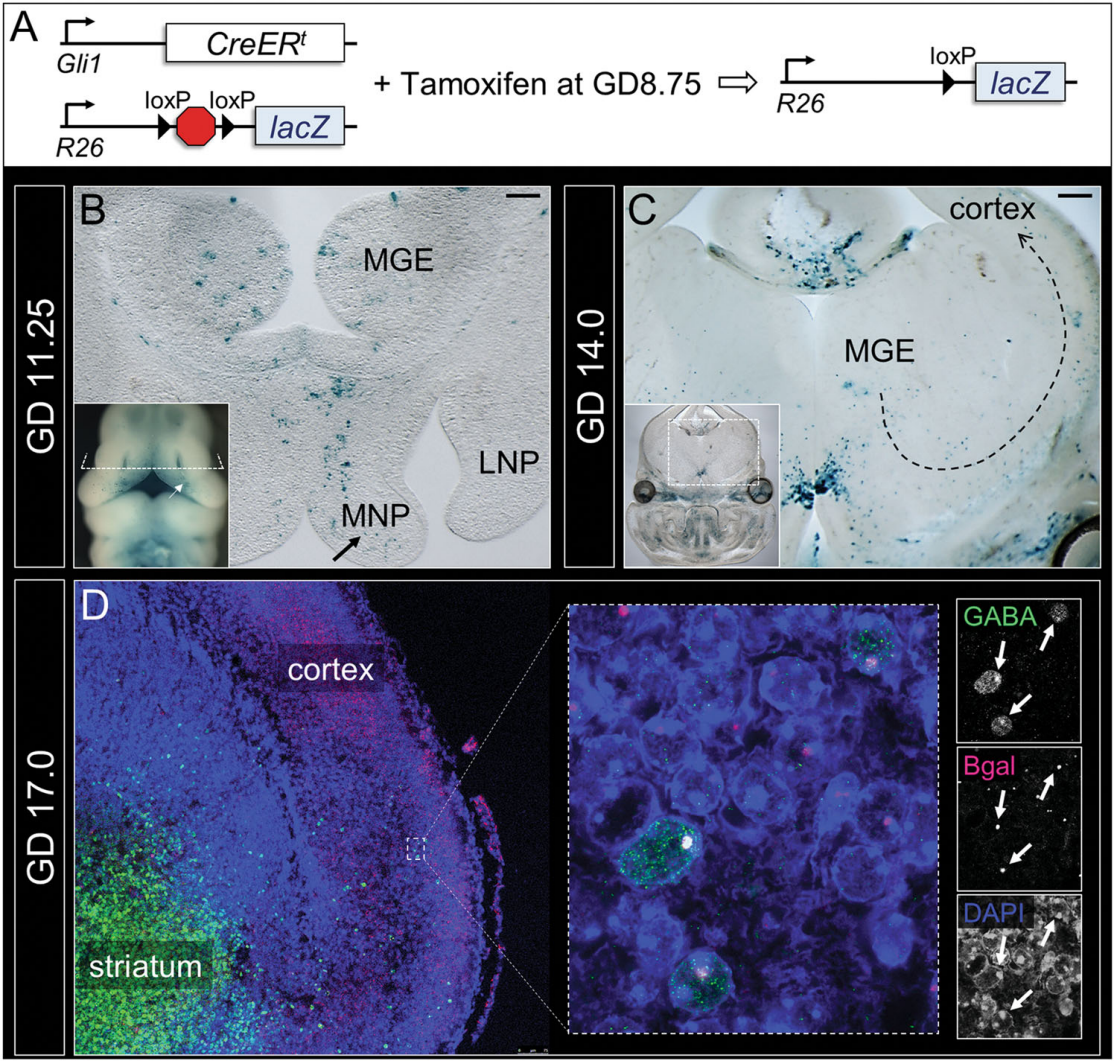
Orofacial and cortical interneuron development occur in spatiotemporal proximity and molecular synchrony. **a** To identify the lineage of SHH ligand-responding cells, mice with *Gli1* promoter-driven and Tamoxifen-inducible Cre recombinase were bred to mice carrying a lacZ reporter with an upstream floxed STOP cassette and administered Tamoxifen at GD8.75. **b** Whole mount (inset) and sectional staining show reporter-positive cell populations in the medial nasal processes (MNPs) (arrows), and adjacently developing MGEs at GD11.25. **c** At GD14.0, reporter-positive populations appear along the presumptive tangential migration path of cINs (dotted arrow). **d** At GD17.0, a population of reporter-positive cells (red) at the deep edge of the cortical plate also express GABA (green). LNP lateral nasal process

We first hypothesized that cells within the developing MGE would be SHH-responsive during the period of sensitivity to OFC formation. To define SHH ligand-responsive cell populations in the developing face and brain, we utilized R26R reporter mice crossed with *Gli1-CreER* ^*t*^ mice^49^. Tamoxifen administration at GD8.75 induced genetic recombination and reporter gene activation in cells expressing the conserved Shh pathway-target gene *Gli1* during the critical period for induction of OFCs (Fig. 2a and S1)^38^. Since reporter gene activation is permanent and retained through cell division, we traced the progeny of these responsive cells in the developing face and brain in a series of cell-mapping experiments. In GD11.25 embryos, reporter-positive populations were present in the medial nasal processes (MNPs), which form the medial portion of the upper lip and primary palate after fusing bilaterally with the paired maxillary processes. (Fig. 2b inset and S1). Sectional analysis revealed additional reporter-positive populations in the MGEs of the developing forebrain (Fig. 2b). Later in development, at GD14, reporter-positive populations were observed in the upper lip and palatal shelves (Fig. S1), in the MGEs, and also along the presumptive tangential migratory path of cINs from the MGE to the cerebral cortex, and within the cerebral cortex itself (Fig. 2c). At GD17, reporter-positive populations were observed in the cortex aligned at both surfaces of the cortical plate but concentrated along the deep side. Dual immunofluorescence staining showed that reporter gene expression co-localized with the cIN marker GABA in a subset of cortical cells (Fig. 2d). This finding demonstrates that GABAergic cortical neurons present in the fetal brain at GD17 are derived from SHH-responsive progenitors found in the MGE that develop in molecular and spatiotemporal concurrence with the upper lip and palate.

### Disrupted MGE development parallels OFC pathogenesis

**Fig. 3:**
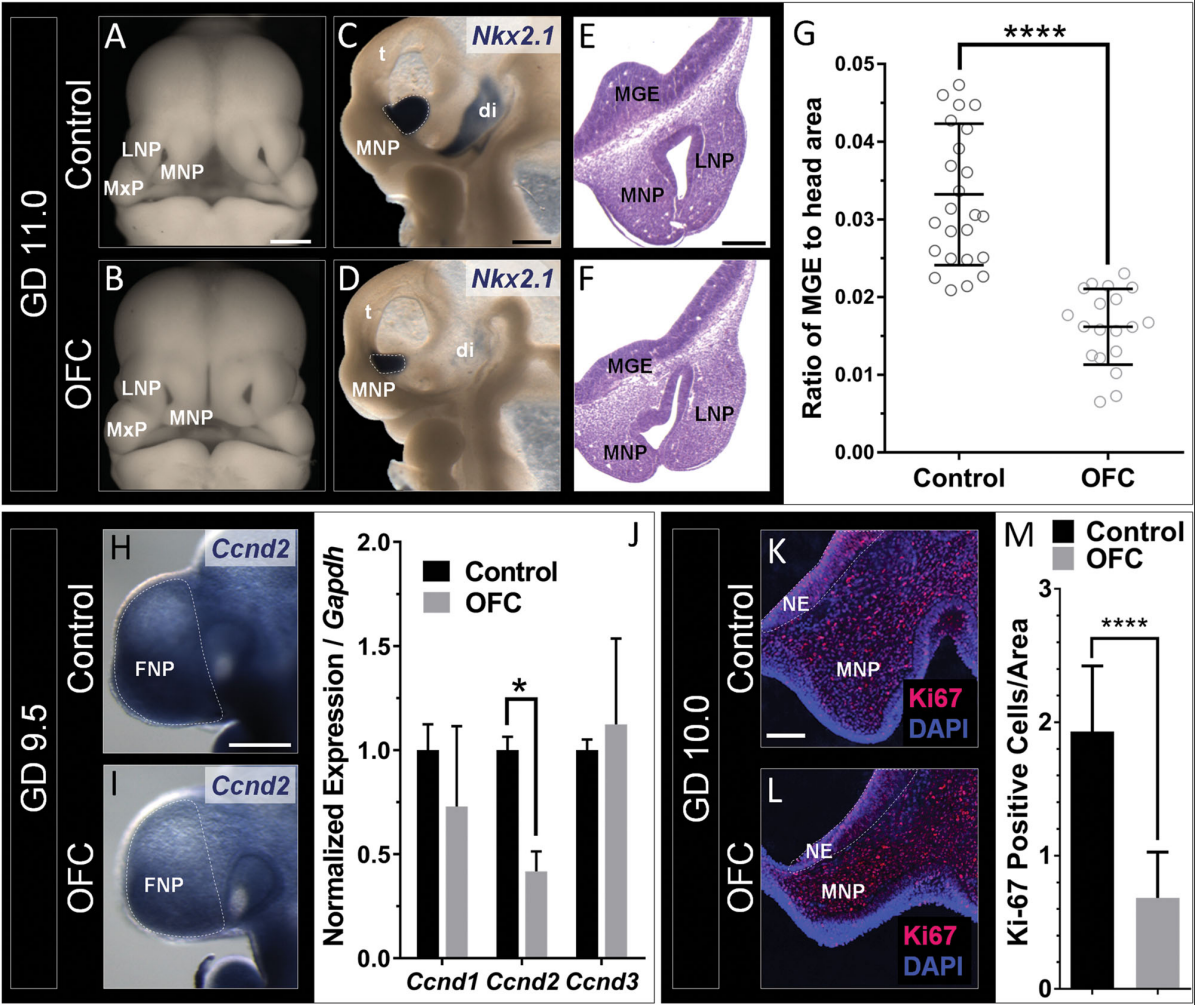
Disrupted MGE development parallels OFC pathogenesis. Deficiency of the MNPs and MGEs in the OFC group is shown in whole embryos (**a**,**b**) and hemisected embryos stained for *Nkx2.1* (**c**,**d**). **e**,**f** The anatomical relationship and paralleled hypoplasia of the MNPs and MGEs is shown by histological staining of transverse sections. **g** Quantification of MGE area relative to total head area (Fig. S2) was calculated from images depicted in **c** and **d**. Individual data points are shown with mean±s.d. of *n* = 24 embryos from three litters for the control group and *n* = 19 embryos from three litters for the OFC group; ****P ≤ 0.0001, two-tailed Student’s *t-*test. Reduced expression of *Ccnd2* in the OFC group is shown by ISH staining (**h**,**i**) and RT-PCR analysis of microdissected FNP tissue of GD9.5 embryos (**j**). Values represent mean±s.e.m. of *n* = 3 pooled litters per group; *P ≤ 0.05, two-tailed Student’s *t-*test. Staining on frontal sections through the neuroectoderm (NE) of GD10.0 embryos (**k**,**l**) demonstrated a reduction in the number of Ki67-positive cells per total area in the OFC group. Values represent the mean±s.e.m.; ****P ≤ 0.0001, two-tailed Student’s *t-*test. LNP lateral nasal process, MNP medial nasal process, t telencephalon, di diencephalon

We next examined cIN development during OFC pathogenesis. GD11.0 embryos were stained by in situ hybridization for *Nkx2*.*1*, which marks the MGEs^50^. In addition to the MNP deficiency that results in cleft lip^40^, cyclopamine-exposed embryos exhibited significant hypoplasia of the MGEs (Fig. 3a–g), suggesting some degree of temporal overlap in the sensitivity of these specific cell populations in the developing face and brain. To test sensitivity of the MGEs during the precise critical window for cleft lip, pregnant dams were administered the potent synthetic Shh pathway inhibitor vismodegib at GD8.75—an exposure paradigm that results in OFCs or “subcleft” phenotypes^38^. Acute vismodegib exposure replicated the MGE deficiency caused by cyclopamine, even in embryos without overt clefts (Fig. S2).

In the developing cerebellum, SHH drives proliferation of neuronal progenitor cells via a MAPK-independent upregulation of D-type cyclins^51^. Suggesting a similar relationship in MGE expansion, we found that expression of *Ccnd2* was significantly reduced in the frontonasal prominence/prosencephalon of cyclopamine-exposed embryos with presumptive OFCs (Fig. 3h–j). Downregulation of *Ccnd2* was relatively specific as no significant effect was observed on the expression of *Ccnd1* or *Ccnd3*. We next examined proliferation 12 h later in the region of neuroepithelium that gives rise to the MGE (Fig. S3). GD10.0 embryos exposed to cyclopamine exhibited a significant reduction in the percentage of Ki67-positive cells relative to vehicle-exposed controls (Fig. 3k–m). These data suggest that OFCs and cIN abnormalities can result from a common cellular mechanism initiated by a single insult.

### OFCs co-occur with altered cIN migration

**Fig. 4:**
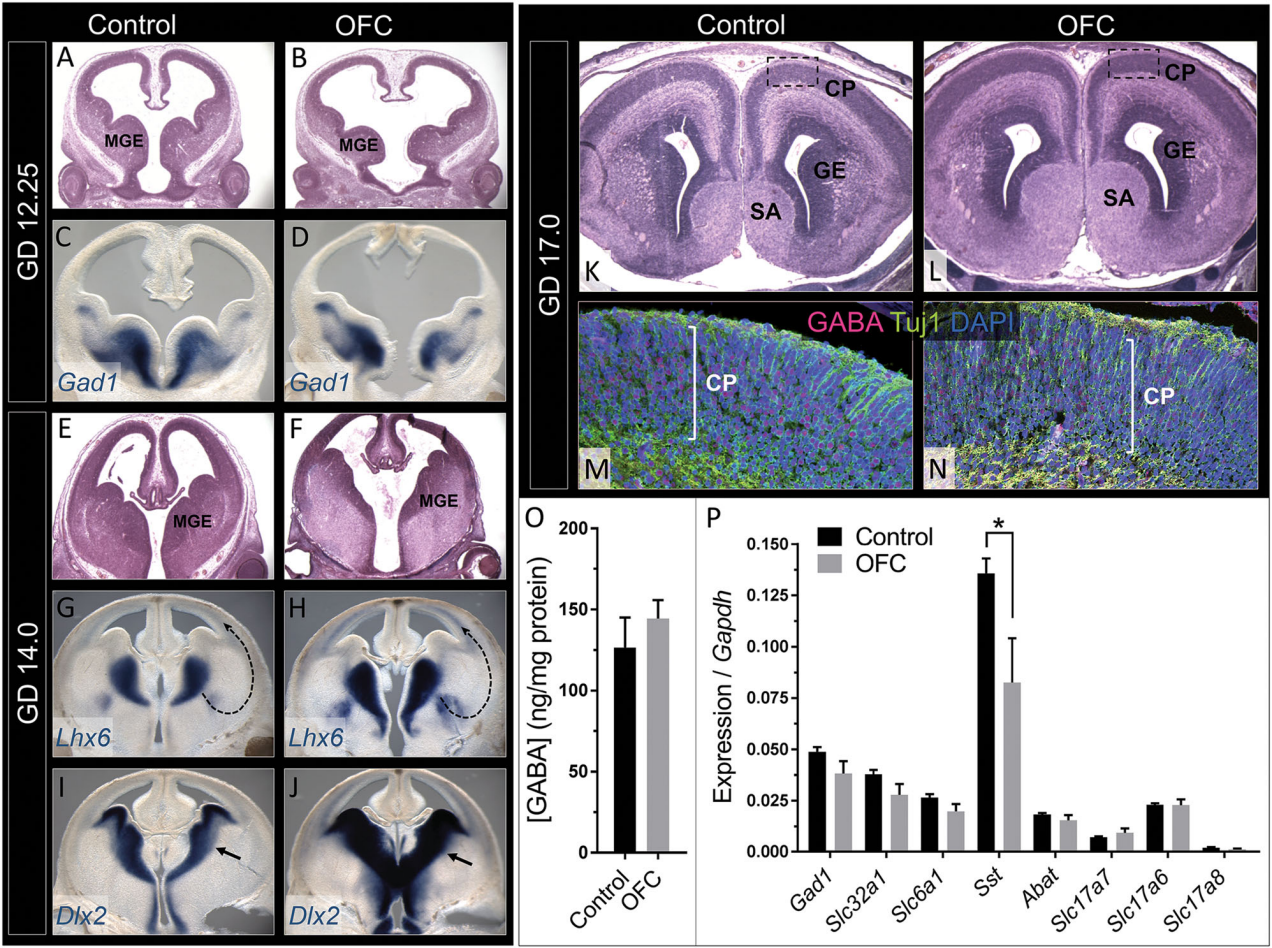
OFC pathogenesis co-occurs with disrupted cIN migration and specification. At GD12.25, the MGEs in the OFC group are dysmorphic (**a**,**b**) but retain expression of the GABAergic precursor marker *Gad1* (**c**,**d**). By GD14.0, differences in MGE morphology in the OFC group are less apparent (**e**,**f**) but the domains of expression of *Lhx6* and *Dlx2* are abnormally expanded (arrows; **g**–**j**). At GD17.0, no major differences are observed between groups in cortical plate histology (**k**,**l**) or in the spatial distribution of GABA-positive cells (**m**,**n**). **o** GABA levels measured in cortical homogenate relative to total protein. Values represent the mean±s.e.m. of *n* = 5 fetuses from two litters for the control group and *n* = 7 fetuses with overt upper lip clefts from two litters for the OFC group; *P ≤ 0.05, two-tailed Student’s *t-*test. **p** RT-PCR analysis of cortical mRNA levels of GABAergic (*Gad1, Slc32a1, Slc6a1, Sst*, and *Abat*) and glutamatergic (*Slc17a7, Slc17a6*, and *Slc17a8*) markers from the cortex at GD17 illustrates a specific reduction of *Sst* in the OFC group. Values represent the mean ± s.e.m. of *n* = 5 fetuses from two litters for the control group and *n* = 7 fetuses with overt upper lip clefts from two litters for the OFC group; *P ≤ 0.05, two-tailed Student’s *t-*test). SA septal area, GE ganglionic eminence, CP cortical plate

We next investigated whether MGE hypoplasia leads to sustained perturbation of cIN development in embryos and fetuses with OFCs. GD12.25 embryos with OFCs exhibited considerable dysmorphology of the subpallial region that contains both the medial and lateral ganglionic eminences, along with increased mid-ventral forebrain width and third ventricular expansion. At GD12.25, *Gad1* is normally expressed in cortical and striatal GABAergic progenitors that occupy the ganglionic eminences. While the subpallium was noticeably dysmorphic, the domain of *Gad1*-expression was grossly normal in embryos with OFCs (Fig. 4a–d).

Beginning around GD13 in the mouse, postmitotic cIN precursors exit the subpallial proliferative zones and undergo tangential migration to the overlying cortex. We investigated interneuron migration by staining for *Lhx6*, a LIM homeodomain transcription factor that regulates cIN migration, and *Dlx2*, a distal-less homeobox transcription factor expressed in subpallial region, progenitors, and migrating interneurons. Histological analysis revealed that early subpallial dysmorphology in embryos with OFCs diminishes as development progresses; as compared to earlier stages, the MGEs in GD14.0 animals appeared less hypoplastic (Fig. 4e–f). Unexpectedly, the spatial expression domains of both *Lhx6* and *Dlx2* were markedly expanded in animals with OFCs (Fig. 4g–j), especially in regions of the subpallium corresponding to the presumptive pathway of cIN migration from the MGE, and along the ventral aspect of the developing cortical plate. While it is unclear whether this reflects precocious differentiation or abnormal migration, these data demonstrate that early perturbation in MGE morphogenesis that accompanies OFC pathogenesis has a sustained impact on cIN development.

### Expression of the cIN subtype-specific peptide Somatostatin is reduced in perinatal mice with OFCs

To examine the consequences of persistent abnormalities in cIN development, we examined the cortical GABAergic system in perinatal mice with OFCs. By GD17, mice with OFCs exhibited expansion of the forebrain septal region as previously observed^24^, while cortical layering and cytoarchitecture appeared approximately normal (Fig. 4k, l). By immunofluorescence, the spatial domain of GABA expression appeared approximately normal in mice with OFCs (Fig. 4m, n). Consistent with the unperturbed spatial distribution, no differences were detected in the total cortical concentration of GABA (Fig. 4o). These results suggest that the increased expression of *Lhx6* and *Dlx2* observed at GD14.0 does not result in an overt overproduction of cortical GABAergic interneurons. We next examined GABAergic subtype-specific mRNA markers in cortical homogenate from GD17 fetal mice. Relative to vehicle controls, animals with OFCs exhibited significantly decreased expression levels of *Somatostatin* (*Sst*), which encodes a cIN subtype-specific peptide. Differences in *Sst* expression appeared specific as additional markers of GABAergic cIN (*Gad1*, *Slc32a1*, *Slc6a1*, and *Abat)* and glutamatergic (*Slc17a7, Slc17a6*, and *Slc17a8*) cell populations were not changed (Fig. 4p). These data suggest that early perturbations in MGE development in mice with OFCs results in a specific disruption in the subset of GABAergic cINs that express *Sst*.

## Discussion

This study identifies molecular and cellular bases that mechanistically link development of the brain and face, and establishes the potential for shared disruption by the same developmental insult. These findings further the growing body of evidence that OFC-associated neurobehavioral traits may, at least in part, stem from primary neurodevelopmental disruption^7,24,25,52,53^ and support a model in which these alterations significantly involve the somatostatin-producing subtype of GABAergic cINs. Importantly, cIN dysfunction, altered GABA signaling, and abnormalities in the neocortical excitatory−inhibitory balance are implicated in the pathophysiology of a wide range of disorders that symptomatically overlap with those seen in OFC, such as seizure disorders, neuropsychiatric illnesses, and impairment of complex cognitive tasks including working memory, sensory integration, and language skills^31,33,35,54–58^.

The evidence described herein is the first to directly demonstrate an OFC−cIN relationship, but scrutiny of previous studies provides several lines of supporting evidence. Some degree of overlap between the programs regulating development of the lip, palate, and cINs is suggested by several mutual genetic regulators including *Wnt5a* ^59–61^, *Gad1* ^62,63^, *Vax1* ^64–66^, and *Gabrb3* ^63,67,68^. These cell populations also share inherent sensitivity to environmental insults, including prenatal alcohol exposure^26^. The concurrent impact of alcohol on the developing brain and face has been extensively documented and presents significant overlap with our emerging understanding of the OFC face-brain relationship^69^. In mice, prenatal ethanol exposure can result in OFCs, deficiency of the MGEs, and disruption of cIN migration^70,71^, while in humans, cortical interneuronopathy has been proposed to contribute to the cortical dysfunction of fetal alcohol spectrum disorders, which encompasses multiple cIN-associated psychiatric conditions^72^.

This study expands upon mouse model findings that have substantiated the link between OFCs and abnormal structural brain development suggested by studies of patient populations. Neuroimaging of individuals with repaired OFCs identified disproportionate volume reductions of the frontal lobe, subcortical nuclei, and cerebellum^14,15,]^. In addition to these overall size reductions, non-uniform shifts in cerebral and cerebellar volumes have also been found^16^. While making the initial association between OFCs and abnormal neurodevelopment, these studies were limited to adolescent and adult populations where brain plasticity during infancy and childhood may be a confounding factor. However, the premise that primary brain abnormalities develop in concert with OFCs is supported by more recent evidence from genetic and teratogenic mouse models. Adult mice with heterozygous mutations of the OFC-associated *Irf6* gene exhibit enlargement of the frontal cortex but decreased volume of the cerebellum in the absence of overt clefts^25^. Providing a foundation for the mouse model utilized in this study, we demonstrated that OFCs caused by transient inhibition of the Shh signaling pathway are associated with subtle abnormalities in forebrain development^24^. Specifically, we defined hypoplasia of the anterior pituitary and olfactory bulbs that may provide an ontological basis for the well-described small stature and olfaction deficit phenotypes in human cohorts with OFCs^73–76^.

Use of a well-characterized mouse OFC model in these studies allowed us to investigate molecular and cellular mechanisms underlying face−brain relationships. This model was chosen because of its etiological and pathological relevance to human OFCs. Several key members and downstream targets of the Shh pathway (*SHH*, *PTCH1*, *GLI2, FOXE1*, and *FOXF2*) are human OFC genes, and we show here that targeted pathway inhibition in our model alters the expression of more than 10% of all suspected human OFC genes (Fig. 1). Being born with an OFC only raises the risk for certain neurobehavioral and psychiatric traits, and given the etiological complexity of OFCs, these mouse model results may relate only to a subset of affected individuals. However, these findings should prompt further investigation of face−brain phenotypes in additional animal models and in human populations that may reveal etiological variables and physical or molecular biomarkers that help predict patient subpopulations particularly vulnerable to specific neurobehavioral outcomes. Increased understanding of the risk factors and pathogenicity of OFC-associated disruptions in CNS development is necessary to develop targeted therapeutic interventions that could lessen the morbidity of common human birth defects.

## Electronic supplementary material

Supplemental Material
