# Supplementary material for: Common basis for orofacial clefting and cortical interneuronopathy
Source: Transl Psychiatry. 2018 Jan 10;8:8. doi: 10.1038/s41398-017-0057-7 (PMC5802454; doi:10.1038/s41398-017-0057-7)
Supplement: Supplementary file 1 — Supplemental Material [file 41398_2017_57_MOESM1_ESM.pdf]

Fig. S1

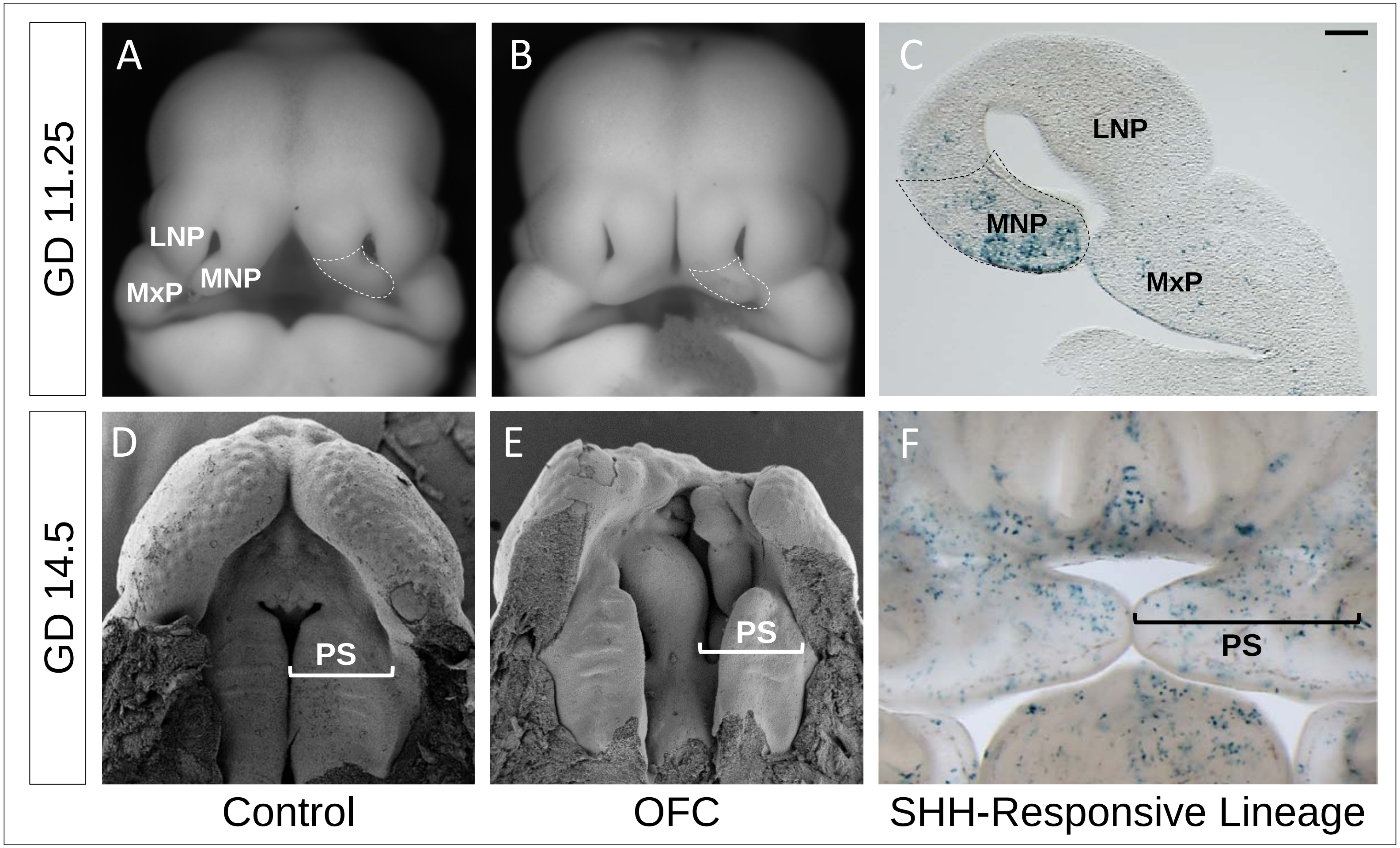

Fig S1. Comparison of dysmorphology and lineage tracing. (A-B) At GD11.25 cyclopamine-exposed embryos exhibit a marked deficiency of the MNP (dashed white outline) that results in cleft lip. (D-E) At GD14.5 cyclopamine-exposed embryos exhibit a deficiency of the secondary palatal shelves (PS) that results in cleft secondary palate. (C,F) Timed-pregnant female B6.129S4-Gt(ROSA)26Sortm1Sor/J mice mated to male Gli1tm3(cre/ERT2)Alj/J males and exposed to a single 50mg/kg Tamoxifen dose by ip injection at GD8.75. β-gal staining shows the lineage of SHH-responding cells in both the MNP at GD11.25 and the palatal shelves at GD14.5, corresponding to tissue deficiency shown in panels B and E.

Fig. S2

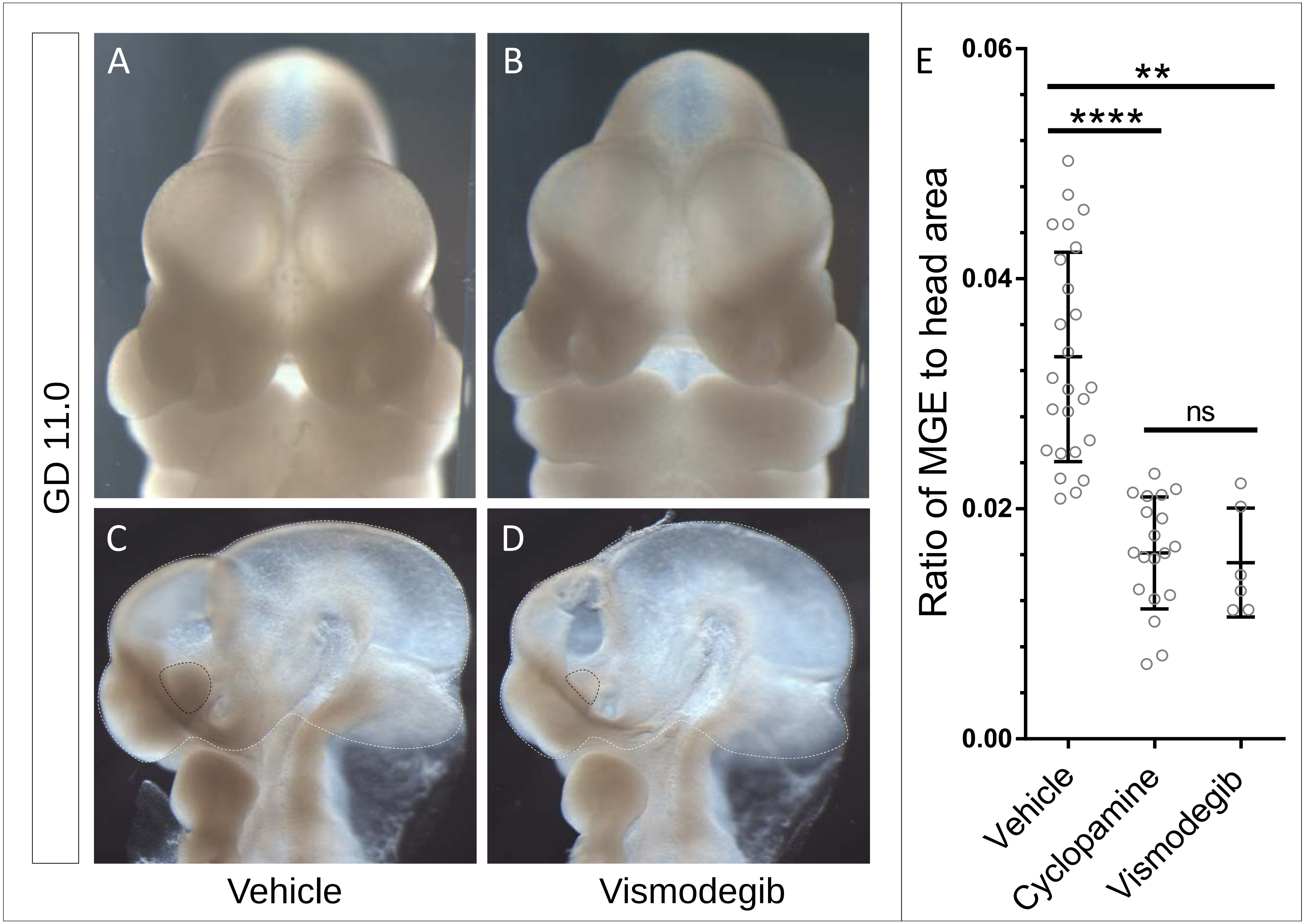

Fig S2. Morphometric analysis of MGE development. Vismodegib (LC Laboratories, Woburn, MA) or vehicle alone was administered at 40mg/kg via oral gavage to pregnant dams at GD8.75 as previously described (36). Unlike cyclopamine-exposed animals with OFCs, the majority of vismodegib-exposed animals do not have overt OFCs (A,B) and are viable into adulthood (n=7/9 survived to P30). Quantification of MGE area (dashed black outline) to total head area (white outline) shows comparable reduction in relative MGE area between cyclopamine and vismodegib-exposed embryos. Values represent mean ± s.d.; ****P ≤ 0.0001 **P ≤ 0.01, 2-tailed Student's t test. PS, palatal shelf; LNP, lateral nasal process; MNP, medial nasal process; MxP, maxillary process.

Fig. S3

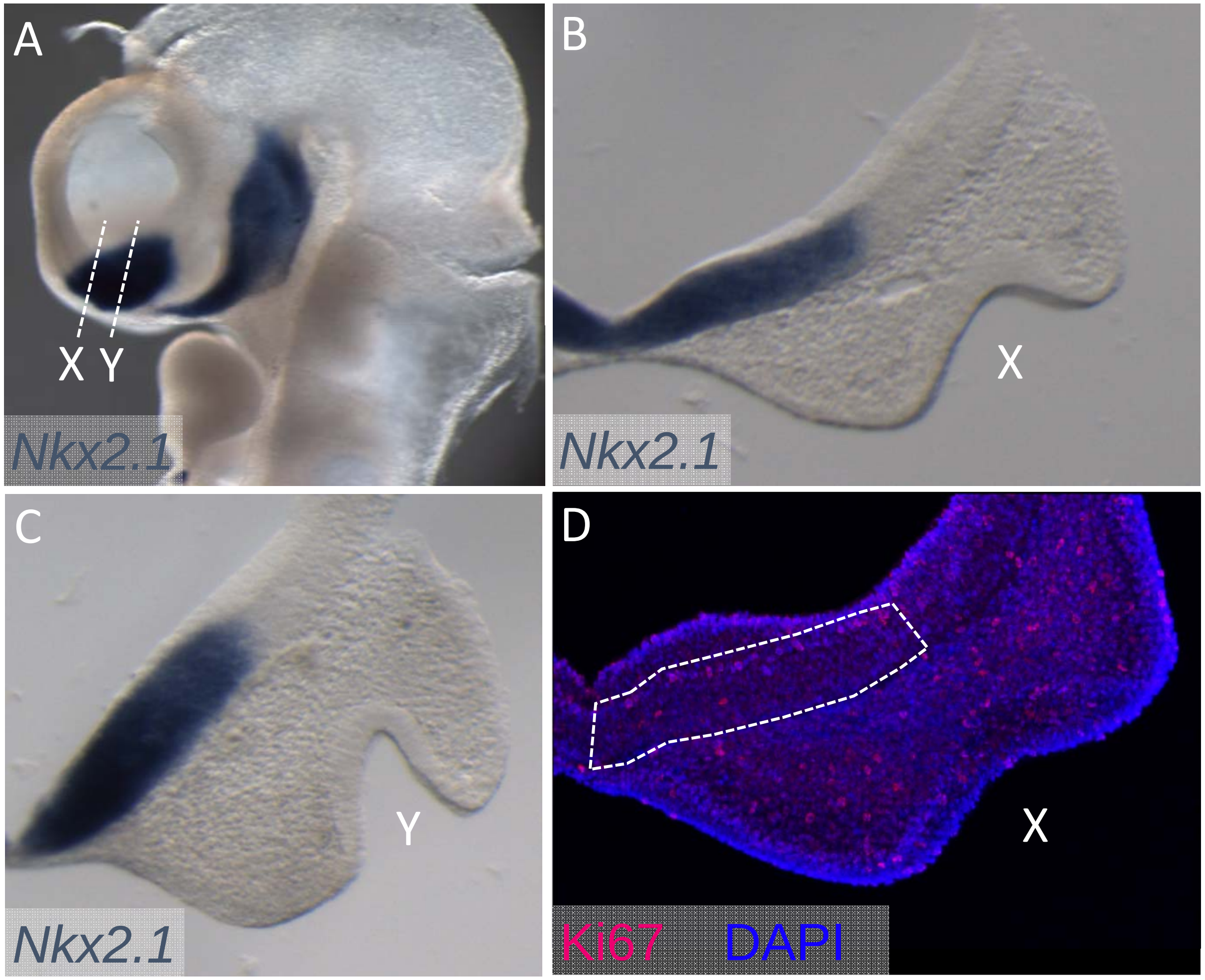

Fig. S3. MGE proliferation assay. (A) A hemisected GD10.0 embryo stained for Nkx2.1 depicts the range of sections (from X to Y) included in the proliferation analysis. (B, C) Sections corresponding to the levels indicated by the lines labeled X and Y in panel A. (D) An example section stained for Ki67 at the plane indicated by X. The dashed outline indicated the area of the neuroectoderm approximating the domain of Nkx2.1 expression and from which Ki67 positive cells were counted.

Table S1

Application	Gene	NCBI Reference Sequence	Forward Primer	Reverse Primer
ISH	*Nkx2.1*	NM_009385.3	TGCGTTTGTCGCTTACAG	AGGAGTTCAGATGGGATAGG
ISH	*Pax6*	NM_001244198.2	AGTGAATGGGCGGAGTTATG	AGTGTGTGTTGTCCCAGGTTC
ISH	*Shh*	NM_009170.3	CCTCTCCTGCTATGCTCCTG	TGTGTGGCACGCTTTATTTC
ISH	*Gli1*	NM_010296.2	CCCTCCTCCTCTCATTCCAC	TCCAGCTGAGTGTTGTCCAG
ISH	*Gad1*	NM_008077.5	AGATAGCCCTGAGCGACGAG	CTCCCACCAACAGCCTTTC
ISH	*Lhx6*	NM_008500.2	AACAGGACAGTCAGCCCAAG	TCTCAGCAGGAGAGGGAAAG
ISH	*Dlx2*	NM_010054.2	AACCACGCACCATCTACTCC	CTGGTCGCCACTTCCAATAC
RT-PCR	*Ccna2*	NM_009828.2	CCGGGCTAGAAGCAAAGTTGTATT	ATGGTGAAGGCAGGCTGTTTAC
RT-PCR	*Ccnb1*	NM_172301.3	CTGCGTCAAATGGTGGCAGATA	ATTTATGCCCACATCCGTGTCC
RT-PCR	*Ccnd1*	NM_007631.2	CATCAAGTGTGACCCGGACTG	CCTCCTCCTCAGTGGCCTTG
RT-PCR	*Ccnd2*	NM_009829.3	GCGGAAAAGCTGTGCATTTAC	TCCACTTCAGCTTACCCAACACT
RT-PCR	*Ccnd3*	NM_007632.2	GTACTCCTGGGTTGCTTTAC	GACTCTCCCTTGCCATTTC
RT-PCR	*Ccne1*	NM_007633.2	CCTCCTCCCAAGTAGTGGGATTAT	AACACAGTGGCACACAGACATAC
RT-PCR	*Gad1*	NM_008077.5	GAGAGGTTGGGTCAGGATCTGTAA	TCCTTCAGTGAGATGGCCTAGATG
RT-PCR	*Slc32a1 (Vgat)*	NM_009508.2	CCTGAAAGGTTGACCGGGAAAT	TACAGTTCCACGACCGGATACA
RT-PCR	*Sst*	NM_009215.1	GCCCAACCAGACAGAGAATGAT	GAAGTTCTTGCAGCCAGCTTTG
RT-PCR	*Slc6a1 (Gat1)*	NM_178703.4	GTCATCATCTCCTGGGCCATCTA	CGCTGGTCATGTTGGTGGTATT
RT-PCR	*Abat*	NM_172961.3	CCTGGCTTTCATGCTTCCTTAGAC	GGGCTGTGATGTGAACTGCTATT
RT-PCR	*Slc17a7 (Vglut1)*	NM_182993.2	TCTCTGAGGAGGAGCGCAAATA	TAGACGGGCATGGACGTAAAGA
RT-PCR	*Slc17a6 (Vglut2)*	NM_080853.3	CCCTGAGGAAACAAGCGAAGAAA	CCTGTGAGGTAGCACCGTAAGA
RT-PCR	*Slc17a8 (Vglut3)*	NM_182959.3	AAGACGGAGTGGAGACAACAGA	GCTGAGGTGAAGCCAGACATTTAG

Table S1. Primer Sequences for real time RT-PCR and ISH. Primers for ISH probe generation (Nkx2.1, Pax6, Shh, Gli1, Gad1, Lhx6, and Dlx2) were designed using Primer3 and the indicated NCBI reference sequence. A 26 nucleotide sequence consisting of a 5bp leader sequence followed by the T7 RNA polymerase recognition sequence (ACGATGTTAATACGACTCACTATAGGG) was added to the 5' end of the reverse primer. Primers for RT-PCR (Ccna2, Ccnb1, Ccnd1, Ccnd2, Ccnd3, Ccne1, Gad1, Slc32a1, Sst, Slc6a1, Abat, Slc17a7, Slc17a6, and Slc17a8) were designed using PrimerQuest and the indicated NCBI reference sequence. All primer sequences were checked for specificity using NCBI Primer BLAST.
